# A Current Perspective on the Potential of Nanomedicine for Anti-Tuberculosis Therapy

**DOI:** 10.3390/tropicalmed8020100

**Published:** 2023-02-03

**Authors:** Khushboo Borah Slater, Daniel Kim, Pooja Chand, Ye Xu, Hanif Shaikh, Vaishali Undale

**Affiliations:** 1 School of Biosciences, Faculty of Health and Microbial Sciences, University of Surrey, Guildford GU27XH, UK; 2 Department of Pharmacology, Dr. D. Y. Patil Institute of Pharmaceutical Sciences and Research Pimpri, Pune 411018, India; 3 Clinical, Assessment, Regulatory and Evaluation (CARE) Unit, International Vaccine Institute, Seoul 08826, Republic of Korea

**Keywords:** tuberculosis, nanomedicine, nanotechnology, *Mycobacterium tuberculosis*, macrophages

## Abstract

Tuberculosis (TB) is one of the ten infectious diseases that cause the highest amount of human mortality and morbidity. This infection, which is caused by a single pathogen, *Mycobacterium tuberculosis*, kills over a million people every year. There is an emerging problem of antimicrobial resistance in TB that needs urgent treatment and management. Tuberculosis treatment is complicated by its complex drug regimen, its lengthy duration and the serious side-effects caused by the drugs required. There are a number of critical issues around drug delivery and subsequent intracellular bacterial clearance. Drugs have a short lifespan in systemic circulation, which limits their activity. Nanomedicine in TB is an emerging research area which offers the potential of effective drug delivery using nanoparticles and a reduction in drug doses and side-effects to improve patient compliance with the treatment and enhance their recovery. Here, we provide a minireview of anti-TB treatment, research progress on nanomedicine and the prospects for future applications in developing innovative therapies.

## 1. Introduction

Tuberculosis (TB) disease is a long-standing scourge of humanity that has plagued mankind for centuries and remains a global epidemic, despite the availability of effective treatment [1,2,3,4]. Caused by the pathogen *Mycobacterium tuberculosis* (Mtb), TB is the second most deadly infectious disease after COVID-19, which causes the deaths of 1.6 million people every year [5,6]. In 2021, around 10.6 million people fell ill with TB around the world. Tuberculosis is also the world’s leading killer of HIV (human immunodeficiency virus)-infected people and a major cause of death arising from antimicrobial resistance. 

Drug-resistant cases, including multi-drug resistance (MDR)-TB and extensively drug-resistant (XDR)-TB cases are emerging global public threats that make the treatment and cure of this disease problematic. Relapse cases with acquired resistance have been observed for the drugs used in the current treatment regimen [7,8]. Mixed infections with multiple strains of Mtb that are antibiotic-susceptible or -resistant is one of the underlying causes of reinfection in drug-resistant TB [9,10]. The detection of drug-resistant cases still requires the development and application of effective antimicrobial-susceptibility testing techniques, which can be applied universally. There is also an urgent need to improve the treatment and find new vaccines and drugs in order to tackle the global health crisis of drug-resistant TB. New research on the development and use of better tools and technologies for TB diagnosis and treatments need to be accelerated for achieving breakthroughs. A holistic approach encompassing prevention, improved and effective detection, surveillance, effective treatment, control and care is needed to end the TB epidemic.

## 2. Current Anti-TB Drugs and Treatment

*Mycobacterium bovis* bacillus Calmette–Guérin (BCG), first administered in 1921, is the current vaccination used for TB prevention [11,12]. The BCG vaccine has a protective efficacy of 60–80% against TB in children, but it is ineffective against pulmonary TB in adults [13,14]. The WHO recommends drug treatments for TB in adults instead of BCG vaccination, which is only effective in children; in addition, the efficacy has also been reported to vary geographically [13,14]. Furthermore, since BCG is a live vaccine, it may cause disseminated infection in immunocompromised individuals [14]. Because of these limitations, there is an urgent need to develop new vaccines.

**Figure 1:**
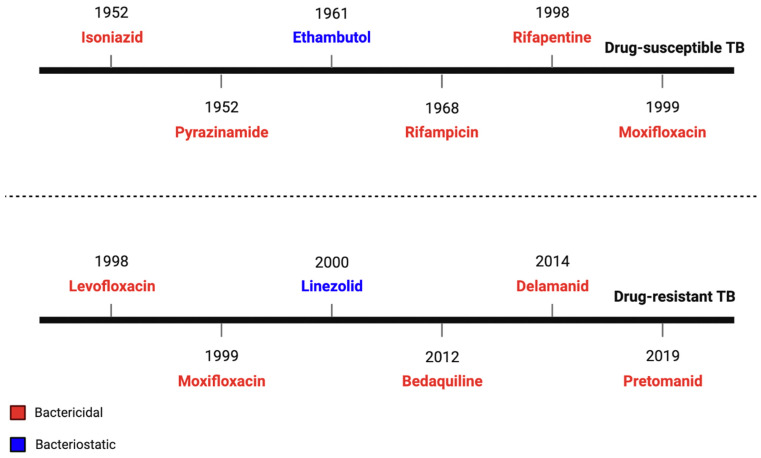
Drugs used for TB treatment. The timeline is non-exhaustive as it only contains the most common and relevant antibiotics for the treatment of drug-susceptible and drug-resistant TB, in accordance with the WHO guidelines (ISBN: 978-92-4-004812-6, ISBN: 978-92-4-000704-8). Bactericidal antibiotics (red) are lethal drugs that inhibit either cell-wall synthesis, protein synthesis, or nucleic-acid synthesis. Bacteriostatic antibiotics (blue) are non-lethal drugs that inhibit bacterial growth and replication.

The timeline of anti-TB drugs that have been approved and are used for treatment is shown in Figure 1. The treatment of newly diagnosed patients remained identical to its initial implementation in the 2010 guidelines, which recommend a 6-month isoniazid (H) and rifampicin (R) regimen, with the addition of pyrazinamide (Z) and ethambutol (E) in the initial 2-month phase (2HRZE/4HR) [15]. The purpose of the long duration of TB treatment is to prevent the relapse of infection. *Mycobacterium tuberculosis* is a slow-growing pathogen with a doubling time of days. The drugs kill Mtb when it replicates, which takes days. The negative outcomes of TB treatment are its serious side-effects, such as hepatotoxicity, rash, gastrointestinal intolerance and neuropathy associated with the multiple drugs used, as well as its long duration [16,17,18,19,20]. These toxicities negatively affect patients and result in non-compliance with and the discontinuation of the treatment, which can ultimately result in the relapse of infection and the development of drug-resistant TB. To circumvent the problems associated with drug toxicities, an updated recommendation comprising a 4-month regimen of isoniazid, rifapentine, moxifloxacin, and pyrazinamide for patients aged 12 years or older with drug-susceptible pulmonary TB was recommended; this regimen did not demonstrate inferiority when compared to the standard-of-care (2HRZE/4HR) regimen [15,21]. 

A successful treatment will clear out actively replicating Mtb and persistent Mtb that are metabolically dormant and non-replicating. Out of the four front-line drugs, rifampicin has the highest sterilizing potential to eradicate persistent bacteria [22,23]. Rifampicin’s sterilizing potential is dose-dependent; the higher concentrations of this drug can enhance sterilization and shorten the treatment duration. However, an optimal high dose of rifampicin with minimal side-effects still needs to be established from clinical trials. A trial conducted on Cornell murine models of TB found that a rifampicin dose ≥30mg/kg in combination with isoniazid and pyrazinamide shortened the treatment and prevented relapse [24]. A phase II trial was conducted in patients recruited in South Africa and Tanzania and it verified that a dose of 35mg/kg rifampicin was safe and promising for shorter treatment regimen [25]. In addition to rifampicin, fluoroquinolones have shown significant potential for use in an improved regimen, but the clinical suitability and the side-effects associated with high doses still need to be determined [25,26]. 

Resistance to the current anti-TB drugs has been a continually emerging problem, and there is extensive ongoing research to combat antimicrobial resistance. The current treatment regimen for drug-resistant TB is lengthy and complicated and has drug-associated toxicities [27]. The regimen is patient-tailored to achieve maximum success. According to the WHO guidelines, the regimen for treating and managing MDR-TB includes an intensive phase, with a combination of four oral drugs and a continuation phase, with at least three drugs [5,6,26,27,28]. In 2022, the WHO released a rapid communication on a novel 6-month treatment regimen in patients with MDR/pre-XDR TB aged 15 years or older. The treatment comprises of bedaquiline, pretomanid, linezolid and moxifloxacin; the latter antibiotic is excluded if drug-susceptibility testing (DST) demonstrates fluoroquinolone-resistant TB (pre-XDR TB patients). This regimen replaced the longer regimens for MDR-TB patients with no previous exposure to the regimen’s drugs. Alternatively, an all-oral 9-month regimen consisting of bedaquiline, fluoroquinolones and linezolid replaced previous, longer bedaquiline-containing regimens. 

The current anti-TB drug regimen is oral and injectable and is well-established, but the rise in MDR-TB and XDR-TB cases suggests that these methods of drug delivery have varying rates of success. To tackle MDR-TB and XDR-TB, there is a need to develop novel drug-delivery systems that can overcome the limitations of the current treatment regimen [26,27,28,29,30]. The intramuscular injection of drugs is a painful route of administration, while drugs administered orally have associated toxicities and negatively affect patient compliance with treatment [26,31]. Recently, new methods, such as inhalation therapy, have received considerable research interest; these therapies have shown comparable efficacy to the existing drug-delivery treatment regimen [32,33,34]. The advantages of inhalation therapy over oral and injectable delivery are reduced doses, reduced toxicities, shorter treatment times, improved patient compliance and the direct administration of the drugs to the site of infection, such as the alveolar macrophages harboring TB bacilli [34]. Several novel drug-delivery systems, including nanoparticles, microparticles, liposome, niosomes, biodegradable microspheres and nanocapsules, are currently undergoing research for replacing the current administration of drugs in their free form. 

## 3. Nanomedicine in TB

Nanomedicine technology includes the engineering and application of nanoparticles, which are colloidal particles with a size of less than one micron and are made of synthetic, semi-synthetic or natural polymers. Drugs are trapped or encapsulated in the polymer matrix as particulates enmesh or solid solution; alternatively, they are bound to the nanoparticle’s surface by adsorption or chemical reaction [31,35]. Nanoparticles target specific sites of delivery and have systemic and controlled drug delivery [36]. This technology improves the efficacy of drugs by improving their poor solubility, circulation and biodistribution. Nanomedicine technology has been successful in circumventing drug resistance in a number of animal and in vitro models of diseases. The in vitro resistance of human and murine cancer cells to doxorubicin was eliminated by the use of doxorubicin-loaded nanospheres [37]. Polyethylene glycol (PEG)- and tobramycin-coated nanoparticles have demonstrated improved penetration through the thick mucus layer and efficient drug delivery for the treatment of cystic-fibrosis patients [38]. There are several studies on the successful application of nanoparticles in antiretroviral therapy for the treatment of HIV infection [39,40]. One study used poly (ethylene oxide)- and poly (epsilon-caprolactone)-modified nanoparticles to incorporate the drug saquinavir and delivered it effectively to THP-1 monocytic/macrophage cells [40]. Anti-HIV agents incorporated in nanoparticles were effective drug-delivery systems that demonstrated improved permeability through the blood–brain barrier for the elimination of HIV from the brain [41]. 

Nanomedicine in TB has gained significant interest in recent years and there have been a number of successful attempts to test the efficacy of these nanomedicines against drug-susceptible Mtb strains in animal models. A TB granuloma is a complex cell construct consisting of Mtb-infected macrophages surrounded by other immune cells, fatty acids and cholesterol. It is challenging to deliver anti-TB drugs to the site of infection in granulomas, which are poorly vascularized [42]. Nanotechnology offers a solution to this problem. Nanoparticles are of sizes comparable to those of intracellular molecules, such as DNA and protein; in addition, they are easily phagocytized by macrophages, allowing the direct targeting of intracellular Mtb in granulomas. 

**Figure 2:**
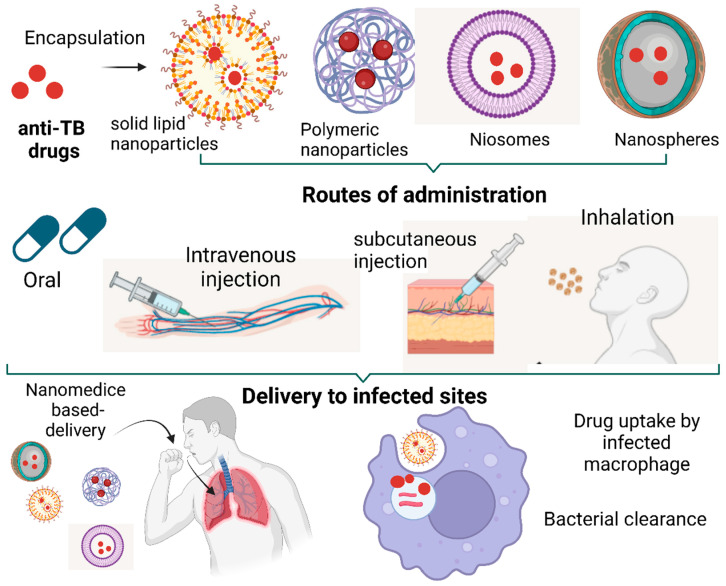
Overview of nanomedicine types, routes of administration and delivery to target sites.

Chaudhury et al. (2022) comprehensively reviewed the advancements in nanomedicine-based-delivery research for treating pulmonary TB [43]. Here, we highlight studies that have shown promising nanotechnology-based treatments in TB and discuss the challenges and future directions of this technology in the next section. A wide variety of polymers have been researched, such as poly (dl-lactide-co-glycolide) (PLG), solid lipid nanoparticles, niosomes and natural polymer alginate (Figure 2) [44,45]. Multiple routes of administration, such as oral, intravenous, subcutaneous and inhalable, have been tested for the administration of nanoparticles and have proved advantageous over the current TB-treatment regimen (Figure 2) [44,46]. Nanomedicine prepared by loading anti-TB drugs (rifampicin, isoniazid and pyrazinamide) onto PLG nanoparticles using the multiple-emulsion technique showed effective bioavailability when administered subcutaneously to mice [47]. Bacterial counts in the lungs and spleens of Mtb-infected mice were significantly reduced compared to anti-TB drugs administered orally [47]. Nanoparticle technology enabled the intravenous injection of anti-TB drugs, which were released directly into the systemic circulation and provided maximum bioavailability [48]. The efficacy of orally administered nanomedicine has also been tested in animal models. Rifampicin, isoniazid and pyrazinamide were encapsulated in PLG nanoparticles and were administered orally to Mtb-infected mice for 46 days [49]. The oral administration of this nanomedicine was effective as it involved reduced doses, increased uptake, biodistribution and complete bacterial clearance from infected organs. This work reported no toxicity in mice from the use of PLG-nanoparticle-based drug delivery. The study also demonstrated a significantly higher sustained delivery of PLG-encapsulated anti-TB drugs that were maintained in blood plasma for up to nine days, whereas the standard free anti-TB drugs were cleared from the plasma within 12 to 24 h. The subcutaneous injection of drugs encapsulated in nanoparticles also showed a sustained delivery of anti-TB drugs that were maintained at a physiologically relevant concentrations for up to 32 days in plasma and 36 days in the lungs and spleen [47]. This research demonstrated that the mean residence time and bioavailability were several-fold higher than those of the unencapsulated free drugs, which were maintained in the plasma and tissues for only up to 12 and 48 h, respectively. The sustained release of drugs offers the potential to lower their doses, which is a major advantage associated with using nanomedicine.

Orally administered nanomedicine proved to be better than the subcutaneous administration of nanoparticles, which reduced bacterial load but did not clear it completely [49,50]. The size of the nanoparticles allowed increased transcytosis through the gut epithelium, which reduced the loss of nanomedicine in the bowels before it entered the bloodstream and, consequently, improved bioavailability [46,51]. Nanomedicine also has the potential to be used for inhalation TB chemotherapy, which has advantages over oral and injectable-drug-delivery systems. Nanomedicine-inhalation therapy can deliver anti-TB drugs directly to alveolar macrophages, which are the primary sites of residence of TB bacilli [46,51]. Upon delivery, macrophages can phagocytize the nanomedicine and directly expose the drugs to intracellular Mtb residing inside the phagosomes for elimination. 

The antimycobacterial properties of nanoparticles have also been explored. Poly (dl-lactide-co-glycolide) nanoparticles with 1,3-β-glucan (GLU) chitosan shells (CSs) loaded with rifampicin were tested for their ability to stimulate host-cell antimicrobial responses against Mtb [52]. The 1,3-β-glucan binds to the macrophage-cell-surface receptor, Dectin-1, which in turn stimulates proinflammatory cytokines, including tumor necrosis factor-α (TNF-α), interferon-γ (IFN-γ) and IL-12 and reactive-oxygen and nitrogen-species production by macrophages. Dube et al. were successful in demonstrating that their nanoparticle, GLU-CS-PLG loaded with rifampicin, enhanced the antimicrobial activity of macrophages [52]. Edagwa et al. also demonstrated the antimycobacterial activity of PLG nanoparticles encapsulated with rifampicin and isoniazid, which showed a 50% reduction in the intracellular growth of *Mycobacterium smegmatis* in monocyte-derived macrophages [53]. Donnellan et al. synthesized solid-drug nanoparticles loaded with rifampicin; this nanoformulation exhibited an antimycobacterial efficacy that was 50-fold higher than that of free rifampicin when tested in an in vitro system [54]. The nanoformulation and mycobacteria colocalized in murine J744A.1 macrophages demonstrated the targeted intracellular delivery of nanoparticles to the site of infection [54]. The drug delivery to intracellular Mtb in macrophages can be targeted by altering the physicochemical and surface characteristics of nanoparticles and by adapting a hybrid nano-approach, i.e., by using polymers that act as ligands and target specific receptors [43]. Mukhtar M et al. demonstrated the promising anti-TB activity of mannosylated-chitosan and hyaolorunic-acid nanoparticles containing isoniazid, which targeted the CD44 and mannose receptors of macrophages [55]. The novel MmpL3 (mycobacterial membrane protein large 3) inhibitors, BM625 and BM819, in nanoemulsion and niosomes, showed potent antimycobacterial activity against Mtb [56].

Nanomedicine appears to be promising for the development of effective therapeutic interventions against drug-resistant TB, since the efficacy of nanomedicine in eliminating drug-susceptible TB infection has been successfully demonstrated by various studies. Increased bioavailability, sustained and targeted delivery to the sites of infection and reduced treatment duration are examples of the potential of nanomedicine, which is promising to eliminate relapses and the emergence of drug resistance.

## 4. Challenges and Future Perspectives

The development of effective anti-TB drugs and vaccines still remains elusive. Several constraints on the current treatment regimen remain, such as high costs, lengthy treatments, toxicity associated with the drugs involved and difficulty in treating MDR-TB, XDR-TB and latent infection. Nanomedicine offers a potential solution for overcoming the challenges of drug delivery to targeted sites associated with conventional TB chemotherapy and to improve the management of drug-resistant TB. Studies using different animal models have demonstrated the efficacy of nanomedicine. These studies reported no toxicity from isoniazid- and rifampicin-encapsulated PLG microparticles on Mtb-infected mice [49,50]. However, no clinical trials have reproduced the efficacies established in animal models [46]. To design an appropriate nanomedicine regime, it is important to determine the toxicological and pharmacokinetic profiles of the nanoparticles and their fate within the human body. The antimicrobial potential of nanoparticles also needs assessment in clinical trials. 

### 4.1. Choice of Nanoparticle Formulations

A number of polymers have been researched for delivering anti-TB drugs in animal models and in in vitro systems [44,46,57,58,59]. Naturally occurring polymer alginate is a potentially superior drug-delivery system to that of other synthetic nanoparticles, such as PLG and polyethylene oxide [44,46]. Alginate is a water-soluble biopolymer and therefore offers drug encapsulation without the need for organic solvents and eliminating any toxicities associated with the solvents. Anti-TB drugs loaded alginate nanoparticles when administered to Mtb-infected mice sustained the drugs in plasma for 12 days and in tissues for 15 days [57,58,59]. This nanomedicine demonstrated complete bacilli clearance in Mtb-infected mice and guinea pigs [58,59]. Further research is needed to identify and formulate new nanoparticles that could demonstrate efficient drug delivery in TB. There is a need to compare existing polymers in order to identify a polymer that offers superiority over others in terms of shelf life, sustained and targeted delivery, bioavailability, vascular permeability, retention at the infection sites and the provision of increased drug lifespan in phagocytes [60,61]. In the case of synthetic polymers, it is crucial to understand the fates of residual polymers in the body, along with their degradation and elimination from the system, as well as to identify any associated toxicological effects. 

The mode of administration of nanoformulation is important. Intravenous administration can lead to accumulation in the bone marrow, spleen, liver and lungs. Even inhaled formulation can enter the vital organs because of their small size; since the toxicity of several nanoparticles is not well established, the therapeutic administration of these nano formulations can produce nanotoxicity in multiple organs. The nanoformulations will also need to be compatible with other drugs when used for the treatment of co-infections and when used in individuals receiving treatment for an existing health conditions, such as diabetes. For instance, TB nanoformulations will need to be compatible when used in parallel with antiretroviral drugs thar are used against HIV to ensure the safe and effective treatment of TB and HIV co-infections. Therefore, further research and clinical validations are still required to verify the use of nanomedicine for TB treatment.

### 4.2. Nanoparticle-Associated Toxicity

Reactive oxygen species (ROS)-mediated cytotoxicity, including oxidative stress, DNA damage, mitochondrial dysfunction and lipid peroxidation, has been reported with several nanoparticles [57]. Aluminum oxide (Al_2_O_3_) nanoparticles caused increased ROS production, the decreased expression of antioxidant genes and locomotive defects in nematode *Caenorhabditis elegans* [62]. Zinc oxide (ZnO) induced cytotoxicity, including increased ROS and cell death, when tested in RAW 264.7 and BEAS-2B cell lines [63]. This study demonstrated the localized dissolution of ZnO in the caveolae of BEAS-2B cells and in the lysosomes of RAW 264.7 cells [63]. Silver (Ag) nanoparticles and carbon nanotubes (CNT) induced increased ROS and mitochondrial dysfunction when tested in in vitro cell models [64,65]. It is therefore crucial to understand the fate of nanoparticles in humans (biological effects, subcellular or organ localization) and, in this regard, rigorous research is needed to eliminate any risks when designing nanomedicine-based drug delivery. 

Multiple factors, such as particle size, shape, surface chemistry and their composition determine the toxicity of these nanoparticles. Clinical translations of such nanoformulations would be challenging, as some of the biological responses, such as the enhanced-permeability and retention (EPR) effect, may not correlate in human subjects [66,67]. Although such polymers are found to be safe in animal-toxicity studies, nanoformulations prepared with these will need to be carefully tested through clinical studies. Additionally, clinical studies of such new nanoformulations for the formation of anti-TB-drug regimens would require a clear safety profile for nanomedicine monotherapy versus combination therapy with other conventional formulations. Additionally, the quantification of nanoparticles is challenging, as the biodistribution of drugs at the tissue level is more complex in human subjects than in small animals.

### 4.3. Commercialization and Accessibility 

For the implementation of nanomedicine in TB chemotherapy, concerted efforts from governments and drug companies are required. Such a venture would relieve the financial burden on current TB-drug production and distribution. Furthermore, additional factors need to be considered in the nanomedicine regimen, such as the availability of nanomedicines in different geographical settings. In developing and underdeveloped countries, populations do not have access to vital medicines and treatment. It is therefore important to identify the most stable and effective polymer that can be used for the production of nanomedicines in various geographical settings and that can be made easily accessible to vulnerable populations. In addition, there are further challenges associated with the operations and logistics for nanomedicine distribution. With the high burden of TB cases in low- and middle-income countries, the distribution of drugs is conducted via programs run by governments or non-government organizations. There is always a high demand for effective drugs and to meet this demand, the assured supply of drugs is critical. The production of nanoparticles is a challenging task in terms of the reproducibility of sizes and polydispersities [66,67]. The capacity to produce standard-quality and large-volume nanoformulations of anti-TB drugs is of high importance to ensure uninterrupted supply for patients.

### 4.4. Innovative Nanomedicine Therapy Targeting Immunometabolism for Host-Directed Therapy in TB

Infection with Mtb induces changes in central carbon metabolism of host immune cells; for instance, in primary human alveolar macrophages and monocyte-derived macrophages, Mtb infection induces a shift in the metabolism of macrophages from oxidative phosphorylation to aerobic glycolysis [68,69,70]. Macrophages infected with Mtb triggered the re-programming of amino-acid metabolism, including arginine and glutamine metabolism and induced the greater production of nitric oxide and the M1-like polarization of macrophages, which is critical for antimicrobial activity [71]. Infection with Mtb induces changes in the function of mitochondria, an organelle that controls the life and death of a cell. During infection, the mitochondrial bioenergetics and membrane potential are perturbed, which helps the pathogenic Mtb to survive in host cells [72,73]. The immunometabolism of macrophages is important for evoking host immune responses to eliminate TB [70,71,74,75]. There is a link between glycolysis and immune-cell responses against Mtb. In mice, the inhibition of glycolysis has been shown to reduce the production of the proinflammatory cytokine, interleukin 1 beta (IL-1β) [74]. Glycolytic glucose metabolism in macrophages is important for limiting bacterial growth and survival through the induction of IL-1β and antimicrobial peptides [70,74]. The knowledge of the immunometabolic phenotype of macrophages in TB currently remains rudimentary. The exploration of immunometabolism and the identification of host mechanisms that can be manipulated for the development of host-directed anti-TB therapies have received increasing research interest.

The potential of nanoparticles in chemo-immunometabolic therapy to modulate metabolism and immune-cells response for improving the outcomes in chronic human diseases has been demonstrated by several studies [76]. A nanoparticle-polymer network prepared using ROS-consuming bond-bridged copolymers with bromodomain-containing protein 4 inhibitor JQ1 (DHCRJ) reduced ROS in tumor cells, reduced glucose uptake for lactate production and facilitated tumor suppression in triple-negative breast cancer [77]. A different nanosystem with 8-Cyclopentyl-1, 3-propylxanthine (DPCPX) (adenosine A1 receptor inhibitor)-encapsulated nanoparticles with PLGA cores and metal-organic-framework shells (with Fe3+/Mn2+–tannic acid) facilitated tumor suppression through the co-inhibition of adenosine A1 receptor and PD-L1 DNAzyme and enhanced dendritic-cell and T-cell responses for anti-tumor activity [78]. Mitochondria-targeting nanomedicine approaches have also been explored for the treatment of human diseases. A ROS-responsive nano-drug delivery system prepared by combining mitochondria-targeting ceria nanoparticles with atorvastatin improved treatment efficacy in acute kidney injury [79]. Nanosystems comprising liposomes showed promising results for the mitochondria-targeted delivery of drugs to treat neurodegenerative diseases [80]. Anticancer drugs and conjugates encapsulated in nanosystems have been designed to target mitochondrial structure and function, such as membrane permineralization, ROS production and apoptosis, to improve cancer treatment [81,82] 

Whilst there has been substantial progress in nanoparticle research for chronic human diseases, such as cancer and neurodegenerative diseases, only limited attempts have been made to examine the immunomodulating properties of nanomedicine as a host-directed therapy for TB. Few studies have explored the use of gallium nanoparticles in anti-TB therapy and demonstrated the efficacy of this approach in vitro [83,84,85]. Macrophages preloaded with gallium (Ga) nanoparticles were demonstrated to have a significantly reduced bacterial burden [83,84]. Gallium nanoparticles modulated cytokine signaling, reduced levels of interleukin-6 (IL-6) and IL-8 secretion for up to 15 days after drug loading and were suggested as effective therapies against HIV and Mtb coinfections [83,84]. An immunotherapeutic liposome comprising phosphatidylserine on the outer membrane and phosphatidic acid in the inner membrane were designed to achieve maximum mycobacterial killing, to promote phagosome maturation and to avoid the pathology associated with the over-production of pro-inflammatory cytokines in human macrophages [86,87]. Furthermore, the technology used to design nanoparticle-based vaccines in TB has also received attention. Das et al. designed a nanoparticle formulation with TLR-2-ligand-coated nanoparticles loaded with the immunodominant peptide derived from the Acr1 protein of Mtb-targeting dendritic cells [88]. This formulation achieved a reduction in bacterial load in the lungs of infected animals. In addition, BCG-nanocages designed with BCG protoplasts enhanced uptake by antigen-presenting macrophages, which demonstrated better viability and showed similar or stronger memory-like immune responses in Vγ2Vδ2 T cells and CD4+/CD8+ T effector cells compared to live BCG vaccination [89]. 

It is evident that nanotechnology has the potential to improve TB treatment and further research in this area will advance clinical applications. The potential of nanoparticles to tune biological processes, such as mitochondrial function, antioxidant processes and inflammatory responses, needs to be established for designing anti-TB therapies. If a nanoparticle formulation can modulate host-cell responses, such as by boosting immunometabolism to mount appropriate immune responses against the pathogen, then this avenue can be explored to design potential host-directed therapies. The ability of nanoparticles to localize within subcellular cellular compartments, such as in mitochondria and lysosomes, could provide site-targeted drug delivery for effective bacterial clearance. 

## 5. Conclusions

Tuberculosis remains one of the biggest infectious killers and there is an increasing problem of drug resistance, which makes the treatment and cure of this disease challenging. There is an urgent need to develop effective drugs with shorter treatment regimens, reduced toxicity and longer bioavailability to combat this drug resistance. The use of nanotechnology and nanomedicine is an attractive avenue that can circumvent the limitations of current TB treatment regimen. This technology offers a slow, sustained and controlled release of anti-TB drugs and provides advantages of low doses, reduced side-effects and improved patient compliance. The ability of nanoparticles to modulate host-cell immunometabolism and responses is currently under-investigated and research in this area could potentially provide new methods to design host-directed therapeutics. In conclusion, nanomedicine offers the potential to accelerate the progress towards eradicating TB and the success of this technology depends on future research and clinical studies.
